# Correlation of Phenotype–Genotype and Protein Structure in *RYR1*-Related Myopathy

**DOI:** 10.3389/fneur.2022.870285

**Published:** 2022-05-26

**Authors:** Xingzhi Chang, Risheng Wei, Cuijie Wei, Jieyu Liu, Lun Qin, Hui Yan, Yinan Ma, Zhaoxia Wang, Hui Xiong

**Affiliations:** ^1^Department of Pediatrics, Peking University First Hospital, Beijing, China; ^2^Department of Biochemistry and Biophysics, Peking University Health Science Center, Peking University, Beijing, China; ^3^Department of Rehabilitation Medicine, Peking University First Hospital, Beijing, China; ^4^Department of Central Laboratory, Peking University First Hospital, Beijing, China; ^5^Department of Neurology, Peking University First Hospital, Beijing, China

**Keywords:** phenotype, genotype, protein structure, *RYR1*-related myopathy, cohort study

## Abstract

**Introduction:**

Next generation sequencing results in an explosive identification of rare variants of *RYR1*, making the correlation between phenotype and genotype complicated. We analyzed the data of 33 patients with *RYR1*-related myopathy, attempting to elucidate correlations between phenotype, genotype, and protein structure of RyR1.

**Methods:**

Clinical, histopathologic, and genetic data were evaluated, and variants were mapped to the cryo-EM RyR1 structure. The three-dimensional structure of the variant on RyR1 was analyzed.

**Results:**

The clinical spectrum was highly variable regardless of the mode of inheritance. Recessive variations were associated with more severe feeding problems and respiratory insufficiency in infancy (*p* < 0.05). Forty pathogenic and likely pathogenic variations were identified, and 14 of them were novel. Missense was the most common variation type regardless of inheritance mode. Arginine (15/45) was the most frequently involved residue. All but one dominant variation clustered in Pore forming and pVSD domains, while recessive variations enriched in Bsol (7/25) and SPRYs (6/25) domains. Analysis of the spatial structure of variants showed that dominant variants may impact RyR1 mainly by breaking down hydrogen or electrovalent bonds (10/21); recessive variants located in different domains may impact the function of RyR1 through different pathways. Variants located in RyR1 coupling sites (PY1&2 and the outermost of Bsol) may cause the most severe clinical manifestation.

**Conclusion:**

Clinical diversity of *RYR1-*related myopathy was impacted by the inheritance mode, variation type, and variant location. Dominant and recessive variants have different sensitive domains impacting the function of RyR1 through different pathways.

## Introduction

Variations in the ryanodine receptor 1 gene (*RYR1*, MIM#180901), encoding the type 1 ryanodine receptor (RyR1), have been recognized as the most common cause of congenital myopathies (1, 2). Both dominant and recessive variations have been reported in *RYR1* (3). Dominant variations have traditionally been associated with central core disease (CCD) and/or a susceptibility to malignant hyperthermia (MHS) (4), while recessive variations predominate in patients with multiminicore disease (MmD) (5, 6), centronuclear myopathy (CNM) (7), and congenital fiber-type disproportion (CFTD) (8). Next generation sequencing has resulted in an explosion in the identification of new *RYR1* variations, expanding the clinical phenotypes of *RYR1-* related myopathies recently (9). Clinical heterogeneity was noted between patients with dominant and recessive *RYR1*-related myopathies, and within patients with the same inheritance mode (9–11). Besides, intra-familial variability was also noted in patients with the same *RYR1* variations (12, 13).

The relationship between phenotype and genotype of *RYR1*-related myopathies is complex and unclear (14). Compared to recessive variations, dominant variations were associated with milder phenotypes in a large cohort (3), while both dominant and recessive variations were associated with severe neonatal-onset phenotypes in another study (15). Hypomorphic variations were associated with severe clinical phenotype and non-central core myopathy in a large combined cohort of recessive cases (16). Changes in protein function may be the underlying cause of clinical heterogeneity. The relationship between the protein function and the spatial structure of RyR1 is not fully understood (17). Different *RYR1* variations impact the function of RyR1 through different pathways, including the RyR1-dependent Ca (2+) releasing and the sarco/endoplasmic reticulum Ca (2+) stores (18). Recently, advances in single-particle cryo-EM have led to the identification of high-resolution structures of RyR1 (19). The complex of RyR1 has been subdivided into many domains including the N-terminal domain (residues 1–627), SPRY1 (residues 628–849), RY1 and 2 (residues 850–1054), SPRY2 (residues 1055–1241), SPRY3 (residues 1242–1656), the junctional selonoid (Jsol) (residues 1657–2144), the bridging selonoid (Bsol) (residues 2145–3613), the central selonoid (Csol) (residues 3667–4174), the transmembrane domain (residues 4541–4956) and the C-terminal domain (residues 4957–5037) (17). According to its conformational changes between different states, the domains of RyR1 were further classified into a cytosolic shell (CS, residues 1–3613) and a channel and activation core (CAC, residues 3614–5037) functional units. The variable phenotype was correlated to the different locations of variants on the protein structure of RyR1 (20).

Considering the increasing new variations, the huge size of the *RYR1* gene and the dominant and recessive mode of inheritance, the clinical interpretation of *RYR1* variations is becoming a huge challenge. In the present study, we retrospectively investigated the clinical, histopathologic, and genetic findings of 33 cases of *RYR1*-related myopathy, and mapped variants onto the cryo-EM structure of RyR1, attempting to analyze the correlations between phenotype, genotype, and the protein structure of RyR1.

## Patients and Methods

### Patients

Thirty-three patients with *RYR1*-related myopathy who were registered from January 2007 to December 2021 (inclusive) in the Department of Pediatrics, Peking University First Hospital were included in this study. Patient inclusion criteria included ①neonatal or early childhood insidious onset, ②slow or non-progressive clinical course, ③variable hypotonia and muscle weakness, ④normal or mildly elevated level of serum CK (creatine kinase), and ⑤confirmed pathogenic or likely pathogenic *RYR1* variations, which have been interpreted as disease-causing according to the American College of Medical Genetics (ACMG) (21). Clinical data of 12 cases have been previously presented elsewhere without variant mapping on RyR1 (22, 23). Open muscle biopsies were performed on 21 patients (21/33), and a pathological diagnosis was made according to the criteria suggested by Dubowitz et al. (24). The present study has been approved by the Ethics Committee of Peking University First Hospital (Beijing, China, approval number: 2018-265), and written informed consent were obtained from all patients and/or their legal guardians (parents) prior to inclusion in the study.

### Clinical Data Analysis

Medical records were retrospectively reviewed. Clinical, pathological, and genetic data were collected. A disease severity rating scale (Supplementary Table 1) was created for patients in this study. Factors in this rating scale included the age at onset, perinatal and neonatal histories (asphyxia, nasogastric tube feeding, and respiratory support), motor development, and orthopedic complications (congenital hip dysplasia or dislocation), and survival. Patients were followed up at our outpatient department. Mobility and musculoskeletal complications were evaluated with physical examinations. Forced vital capacity was measured in patients over 6 years old. Cardiac function was monitored with electrocardiograms and echocardiograms. Intelligence was compared with normal peers without evaluation scales.

### Genetic Analysis

Blood samples were collected, and genomic DNA was extracted from leukocytes. *RYR1* variations were screened through a gene panel or whole exome sequencing strategy. The coding exons of 169 myopathy-causing genes (Supplementary Table 2) were included in the panel (Kangso medical inspection, China; MyGenostics, Beijing). High coverage (≥100×) sequencing is used to detect SNPs (single nucleotide polymorphisms) and indel variations. The following variations are taken into further consideration: (1) marked as a pathogenic variant in the Human Genome Mutation Database (HGMD), (2) nonsense variations, (3) frameshift variations, and (4) located in an essential splice site. The pathogenicity of non-synonymous variation was evaluated by using multiple algorithms, including SIFT, Ployphen-2, Mutation-Taster, PROVEAN, and Splice-Site Prediction by Neural Network. The exomes were captured using the xGen Exome Research Panel v1.0 (Integrated DNA Technologies) and sequenced on an Illumina HiSeq2000 (Illumina, San Diego, CA, USA) with 100-bp paired-end reads (Kangso Medical Inspection, China; MyGenostics, Beijing). After the sequencing, the raw data was saved in a FASTQ format, and then the bioinformatics analysis was performed before being transformed to the VCF format. Variations were further annotated by ANNOVAR and associated with multiple databases, such as 1000 genome, ESP6500, dbSNP, EXAC, Inhouse (MyGenostics), HGMD, as well as predicted by SIFT, PolyPhen-2, MutationTaster, GERP++. Then the potential pathogenic variations were selected in downstream analysis: (i) variation reads should be more than 5, variation ratio should be no <30%; (ii) removing the variations with a frequency of more than 5% in 1000 genome, ESP6500, and Inhouse database; (iii) If the variations existed in InNormal database (MyGenostics), then dropped; (iv) the synonymous variations reported in HGMD were left, and the remaining of the synonymous variations were excluded. All candidate pathogenic variations were confirmed by Sanger sequencing. The interpretation of the variations was made according to the guidelines provided by the American College of Medical Genetics (ACMG) (21).

### Variant Mapping

Variant analysis and graphical representation were performed with Pymol software (version 2.0.4; Schrödinger, LLC, NY) using PDB (Protein Data Bank) structure PDB: 7M6A Primed state (25). All *RYR1* coding-region variants identified in this cohort (*n* = 45) were mapped to the RyR1 monomer based on domain location, except for one splice variation affecting unassigned residue. Variants were further mapped based on clinical severity using the above-mentioned scale. Variants associated with variable phenotypes were mapped in different colors, i.e., red (severity score > 10), blue (severity score 6–10), green (severity score 1–5), and gray (severity score of 0). After multiple cases were associated with a specific variant, an average clinical severity score was calculated.

### Statistics Analysis

All statistical tests were conducted using the Statistical Package for the Social Sciences version 26 (SPSS; IBM, Armonk, NY, USA). For genotype–phenotype comparisons, participants were grouped based on the mode of inheritance AD (autosomal dominant) or AR (autosomal recessive). For structure–phenotype comparisons, participants were grouped based on the location of *RYR1* variations: only in the RyR1 CS domain, only in the RyR1 CAC domain, or both. Descriptive statistics were generated for each group. Clinical severity differences between groups was assessed by an independent *t* test. Fisher's exact test or χ^2^ test was used to compare the occurrence rate of specific clinical phenomena between patients with different inheritance mode.

## Results

A total of 33 patients (18 male, 15 female) with *RYR1*-related myopathy were recruited. Clinical features of 33 patients were provided (Supplementary Table 3), 21 patients with dominant *RYR1* variations, and 12 with recessive variations. Ten out of them had a significant family history, eight were associated with dominant inheritance, and two with recessive inheritance. There was no history of parental consanguinity. Premature birth occurred in one family. No malignant hyperthermia was noted.

Variable generalized muscle weakness and/or motor development delay were noted in all patients, presenting as pronounced generalized hypotonia, weakness, and motor milestone delay. Twenty patients attained independent walking in the age ranging from 13 to 48 months old, respectively. Thirteen patients never acquired independent walking at their last visit, and three of them were <1 year old. The follow-up time of patients ranged from 34 days to more than 40 years (median 5.5 years). Motor ability was stable or improved in most patients, declined in three patients. One patient (Pt 1) attained independent walking at 4 years old and became wheelchair-bound at 17 years old. Two patients died at 34 days and 7 months old, respectively. Muscular complications including variable scoliosis and/or joint contracture appeared in 12 patients during follow-up. All alive patients had normal cardiac function, evaluated using echocardiograms and electrocardiograms. Two patients (Pt 13 and 15) had mildly decreased forced vital capacity; one patient (Pt 30) had lung function failure requiring ventilator support since the last visit. All patients had intelligence comparable to normal peers.

### Clinical Variance Between Patients With Dominant and Recessive Inheritance

The onset age ranged from birth to 2 years (0.52 ± 0.71 y). The neonatal presentation was noted in 11/21 cases with dominant inheritance and 6/12 with recessive inheritance (χ*2* = 0.017, *p* > 0.05). Reduced fetal movements were noted in 12/18 patients with dominant inheritance and 2/11 cases with recessive inheritance (χ*2* = 6.428, *p* < 0.05). Congenital hip dysplasia was noted in 12/21 patients with dominant inheritance and 3/12 with recessive inheritance (χ*2* = 3.182, *p* > 0.05). Variable degrees of motor development delay was noted in all these patients, 11/21 of patients with dominant inheritance attained independent walking at 25.4 ± 12.7 months old, and 9/12 with recessive inheritance attained independent walking at 18.3 ± 6.5 months old. There was no significant difference in motor milestones between patients with dominant and recessive inheritance (*p* > 0.05).

There was no significant difference in the rate of feeding problems between patients with dominant (6/21) and recessive inheritance (5/12) (*p* > 0.05, Fisher's exact test). It seems that patients with recessive inheritance had more severe feeding problems. Tubular feeding in two patients (Pt 12 and 19) with dominant inheritance lasted 1 month, while the other four patients had difficulty sucking with no need for tubular feeding. Four out of five patients with recessive inheritance required continuous tubular feeding, the other without such need. Out of them, two patients' (Pt 28 and 32) nasogastric feeding continued to death, one patient (Pt23) discontinued tube feeding after 3 months, while one patient (Pt30) was still on tube feeding after 1 year since the last visit. The significant difference in respiratory insufficiency was noted in patients with dominant (1/21) and recessive inheritance (4/12) (*p* < 0.05, Fisher's exact test). One patient (Pt 19) with dominant inheritance received ventilator support for 27 days. Four patients (4/12) with recessive inheritance relied on continuous ventilator support. One of them (Pt 23) discontinued ventilator support after 3 months, two of them (Pt 28 and 32) continued ventilator support till death, and one (Pt 32) was still on it after 1 year since the last visit. Two patients with recessive inheritance died before the age of 7 months old, while no patient with dominant inheritance died. Though there was no significant variance between the two groups as to the clinical severity grade (*p* > 0.05), there was more severe bulbar and respiratory involvement in patients with recessive inheritance, which contributed to the early death of the patients.

### Genotype and Phenotype of *RYR1*

A genetic diagnosis was confirmed in all 33 patients. Genetic and pathologic data was provided in Table 1. In total, 40 pathogenic and likely pathogenic variations were identified, and three variations with uncertain significance. Out of them, c.14582G>A, c. 14581C>T, c.14422_14423delinsAA occurred twice in two separate families, respectively, 14 variants were novel (Supplementary Table 4), including 11 pathogenic or likely pathogenic variations *RYR1* c.14650T>C, c.13904A> G, c.14591A>C, c.14811C>G, c.2792 T>C, c.10714C>G, c.13676G>A, c.9623C>T, c.839G>A, c.7614+1>A, and c.9571G>A, and three variations with uncertain pathogenicity, *RYR1* c.12324G>C, c.13762C>T, and c.12732_12743del. One variation c.12324G>C was found in gnomeAD genome EAS database with the frequency of 0.0026, two variations c.13691G>A and c.9623C>T were found in gnomeAD genome ALL database with the frequency of 0.00003187 and 0.00003185, respectively, others were all absent in gnomeAD database. Thirteen patients with dominant inheritance had pathologic diagnosis as CCD, eight patients without pathologic diagnosis, and two of them were presumed as CCD because their father had a pathological diagnosis. Eight patients with recessive inheritance were associated with different pathological changes, including MmD in six cases, CFTD in one case, CNM in one case, and four patients without a pathological diagnosis.

**Table 1:** Genetic and pathologic data of patients.

**Pt**	**Pathology**	**Inheritance**	**Gene variation**	**Variation type**	**Origin**	**Pathogenicity**	**Protein**
1	CCD	AD	c.14596A>G^‡^	missense	*de novo*	P	p. Lys4866Gln
2	CCD	AD	c.7111G>A^‡^	missense	*de novo*	P	p. Glu2371Lys
3^#^	CCD	AD	c.14678 G>A^‡^	missense	Paternal	P	P. Arg4893Gln
4^#^	CCD	AD	c.14741G>C^‡^	missense	Paternal	P	P. Arg4914Thr
5	CCD	AD	c.14447A>G^‡^	missense	*de novo*	P	p. Asp4816Gly
6	CCD	AD	c.14582G>A^‡^	missense	*de novo*	P	p. Arg4861His
7	/	AD	c.14650T>C	missense	*de novo*	LP	p. Tyr4884His
8^*^	CCD	AD	c.G14719A ^‡^	missense	Paternal	P	p. Gly4907Ser
9^*^	CCD	AD	c.14693T>C^‡^	missense	Paternal	P	p. Ile4898Thr
10	/	AD	c.14581C>T^‡^	missense	*de novo*	P	p. Arg4861Cys
11^*^	CCD	AD	c.13904A>G	missense	Maternal	LP	p. Glu4635Gly
12^*^	CCD	AD	c.14591A>C	missense	Paternal	LP	p. Tyr4864Ser,
13^*^	/	AD	c.13909 A>G^‡^	missense	Maternal	P	p. Thr 4637ALa
14	/	AD	c.14422_14423delinsAA^‡^	missense	*de novo*	P	p. Phe4808Asn
15	CCD	AD	c.13913G>A^‡^	missense	*de novo*	P	p. Gly4638Asp
16	/	AD	c.14582G>A^‡^	missense	*de novo*	P	p. Arg4861His
17^*^	/	AD	c.14678G>A^‡^	missense	Maternal	P	p. Arg4893Gln
18^*^	CCD	AD	c.14422_14423delinsAA^‡^	missense	Maternal	P	p. Phe4808Asn
19	/	AD	c.13952A>C^‡^	missense	*de novo*	P	p. His 4651Pro
20	CCD	AD	c.14811C>G	missense	*de novo*	LP	p. Ile4937Met
21	/	AD	c. 14581C>T ^‡^	missense	*de novo*	P	p. Arg4861Cys
22	MmD	AR	c.3880G>T ^‡^	missense	Maternal	LP	p. Val1294Phe
			c.14473C>T^‡^	missense	Paternal	P	p. Arg4825Cys
23^*^	MmD	AR	c.658C>T ^‡^	missense	Paternal	LP	p. Arg220Cys
			c.4715T>C ^‡^	missense	Maternal	LP	P. Met1572Thr
24	MmD	AR	c.4454G>A ^‡^	missense	Paternal	LP	p. Ser1485Asn
			c.3494G>A^‡^	missense	Maternal	P	p. Gly1165Asp
25	CNM	AR	c.2044C>G ^‡^	missense	Paternal	LP	p. Arg682Gly
			c.6823G>A ^‡^	missense	Maternal	LP	p. Val2275Met
26	CFTD	AR	c.12536G>A^‡^	missense	Maternal	P	p. Arg4179His
			c.1675dup^‡^	frameshift	Paternal	P	p. le559AsnfsTer11
27	/	AR	c.3523G>A^‡^	missense	Maternal	P	p. Glu1175Lys
			c.7330C>T^‡^	nonsense	Paternal	P	p. Gln2444Ter
28	/	AR	c.14645 C>T^‡^	missense	Paternal	P	p. Thr4882Met
			c.2792 T>C	missense	Maternal	LP	p. Leu931Pro
29	MmD	AR	c.10729C>G c.13691G>A	missense missense	Maternal Paternal	LP LP	p. Gln3577Glu p. Arg4564Gln
30	/	AR	c.6721C>T^‡^ c.9623C>T	nonsense missense	Paternal Maternal	P LP	p. Arg 2241Ter p. Pro3208Leu
31	MmD	AR	c.12739_12750del c.839G>A	indel missense	Paternal Maternal	Uncertain LP	p. Ala4247_Ile4250del p. Arg280Gln
32^*^	/	AR	c.7614+1G>A c.9571G>A	splice missense	Paternal maternal	P LP	Splicing p. Gly3191Arg
33	MmD	AR	c.14939C>T^‡^	missense	Paternal	LP	p. Thr4980Met
			c.12324G>C	missense	Maternal	Uncertain	p. Gln4108His
			c.13762C>T	missense	Maternal	Uncertain	p. Pro4588Sert

*RYR1 NM_000540.3, Pt, patient;*

* *, positive family history;*

‡ *, previously reported; /, data unavailable; CCD, central core disease; MmD, multiminicore disease; NM, nemaline myopathy; CNM, centronuclear myopathy; CFTD, congenital muscle fiber disproportion; AD, autosomal dominant; AR, autosomal recessive; P, pathogenic; LP, likely pathogenic*.

**Figure 1:**
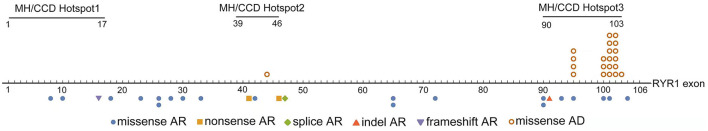
Distribution of variations across the *RYR1* coding region.

The distribution of variations across the *RYR1* coding region was shown in Figure 1. Dominant variations were all located in the MH/CCD hotspot area 3, except one in MH/CCD hotspot 2 area. Recessive variations are distributed across the whole *RYR1* coding region. In patients with recessive inheritance, the two variations were located either completely out of hotspot 3, or with only one allele in hotspot 3. No patients had two recessive variations both located within the hotspot 3 area. Compared to the clinical severity of patients with one allele variation within hotspot 3 and others with no variation in hotspot 3, there was no significant difference (*p* > 0.05).

The consequence of dominant variation c.14422_14423delinsAA (p. Phe4808Asn) was the same as a missense variation, which is classified as missense variation in the present study. Missense variation (41/46, 89.1%) was the most common variation type in all patients. All dominant variations were missense variations, while different types of variations were noted in patients with recessive inheritance mode, including frameshift (*n* = 1), nonsense (*n* = 2), splice (*n* = 1), and indel (*n* = 1) variation (Table 1; Figure 1). Patients with recessive variations had either a combination of a missense and a hypomorphic variation (nonsense, frameshift, indel, or splice) or two missense variations. No patient had two hypomorphic variations. There was no significant difference between the clinical severity of patients with two missense variations and those with a combination of a missense and a hypomorphic variation (*p* > 0.05).

### Phenotype and *RyR1* Structure Analysis

Affected RyR1 domains and changes in protein properties are shown in Table 2. All dominant variations were located in the channel Pore domain (13variations) or Pseudo-voltage-sensor domain (pVSD, seven variations), except one in Bsol. Recessive variations affect SPRY1 (1), SPRY2 (2), SPRY3 (3), Bsol (7), Pore(2), NTD-AB domain (2), TaF (2), Nsol (1), Repeat 1-2 (1), pVSD (2), CTD (1), EF (1) domains [based on the updated structure of RyR1 in rabbit (19)]. The canonical splice variation (c.7614+1G > A) is located adjacent to exon 47, contributing to coding the Bsol domain. So, the location of this variation was attributed to the Bsol domain in the present study. Among recessive variations, the most frequently affected domains were Bsol and SPRYs. According to the role in the gating and activation of RyR1, the domains of RyR1 were divided into two functional units including a cytosolic shell (CS, residues 1–3613) and a channel and activation core (CAC, residues 3614–5037) domain. Dominant variations all affect the CAC domain, except one in Pt 2. While recessive variations involved only CS domain in six patients, only CAC domain in one patient, and both CA and CAC domain in five patients. There was no significant clinical severity variance between patients with recessive variations affecting two domains (CS and CAC) and one domain only (CAC or CS) (*p* > 0.05). There was no significant difference between clinical severity of patients with only CAC involvement and only CS involvement (*p* > 0.05).

**Table 2:** Affected RyR1 domains and changes of protein property (based on the updated structure of RyR1 in rabbit, 19).

**Pt**	**Protein human/rabbit**	**Region of RyR1**	**Change of protein charges**	**Change of protein hydrophilic properties**	**Functional unit**
1	p. Lys4866Gln /Lys4865	Pore	Positive to neutral	Unchanged	CAC
2	p. Glu2371Lys /Glu2371	Bsol	Negative to positive	Unchanged	CS
3^*^	p. Arg4893Gln /Arg4892	Pore	positive to neutral	Unchanged	CAC
4^*^	P. Arg4914Thr/Arg4913	Pore	Positive to neutral	Unchanged	CAC
5	p. Asp4816Gly/Asp4815	pVSD	Negative to neutral	Unchanged	CAC
6	p. Arg4861His /Arg4860	Pore	Unchanged	Unchanged	CAC
7	p. Tyr4884His/Tyr4883	Pore	Neutral to positive	Unchanged	CAC
8^*^	p. Gly4907Ser/Gly4906	Pore	Unchanged	Unchanged	CAC
9	p. Ile4898Thr/Ile4897	Pore	Unchanged	Hydrophobic to hydrophilic	CAC
10	p. Arg4861Cys/Arg4860	Pore	Positive to neutral	Unchanged	CAC
11^*^	p. Glu4635Gly/Glu4634	pVSD	Negative to neutral	Unchanged	CAC
12^*^	p. Tyr4864Ser/Tyr4863	Pore	Unchanged	Unchanged	CAC
13^*^	p. Thr4637Ala/Thr4636	pVSD	Unchanged	Hydrophilic to Hydrophobic	CAC
14	p. Phe4808Asn/Phe4807	pVSD	Unchanged	Hydrophobic to hydrophilic	CAC
15	p. Gly4638Asp/Gly4637	pVSD	Neutral to negative	Unchanged	CAC
16	p. Arg4861His/Arg4860	Pore	Unchanged	Unchanged	CAC
17 ^*^	p. Arg4893Gln/Arg4892	Pore	Positive to neutral	Unchanged	CAC
18^*^	p. Phe4808Asn/ Phe4807	pVSD	Unchanged	Hydrophobic to hydrophilic	CAC
19	p. His4651Pro/His4650	pVSD	Positive to neutral	Hydrophilic to hydrophobic	CAC
20	p. Ile4937Met/Ile4936	Pore	Unchanged	Unchanged,	CAC
21	p. Arg4861Cys/Arg4860	Pore	Positive to neutral	Unchanged	CAC
22	p. Val1294Phe/Val1295	SPRY3	Unchanged	Unchanged	CS
	p. Arg4825Cys/Arg4824	Pore	Positive to neutral	Unchanged	CAC
23^*^	p. Arg220Cys/Arg221	NTD-B	Positive to neutral	Unchanged	CS
	p. Met1572Thr/Met1573	SPRY3	Unchanged	Hydrophobic to hydrophilic	CS
24	p. Ser1485Asn/Ser1486	SPRY3	Unchanged	Unchanged	CS
	p. Gly1165Asp/Gly1166	SPRY2	Neutral to negative	Unchanged	CS
25	p. Arg682Gly/Arg683	SPRY1	Positive to neutral	Unchanged	CS
	p. Val2275Met/Val2275	Bsol	Unchanged	Unchanged	CS
26	p. Arg4179His/Arg4180	TaF	Unchanged	Unchanged	CAC
	p. Ile559AsnfsTer11/Ile560	Nsol	Unchanged	Hydrophobic to hydrophilic	CS
27	p. Glu1175Lys/Glu1176	SPRY2	Negative to positive	Unchanged	CS
	p. Gln2444Ter/Gln2444	Bsol	Unknown	Unknown	CS
28	p. Thr4882Met/Thr4881	Pore	Unchanged	Hydrophilic to hydrophobic	CAC
	p. Leu931Pro/Leu932	RY1&2	Unchanged	Unchanged	CS
29	p. Arg3577Gly/Arg3576	Bsol	Positive to neutral	Unchanged	CS
	p. Arg4564Gln/Arg4563	pVSD	Positive to neutral,	Unchanged	CAC
30	p. Arg2241Ter/Arg2241	Bsol	Unknown	Unknown	CS
	p. Pro3208Leu/Pro3208	Bsol	Unchanged	Unchanged	CS
31	p. Ala4247_Ile4250del/	TaF	Unchanged	Unchanged	CAC
	Ala4248_Ile4251				
	p. Arg280Gln/Arg281	NTD-B	Positive to neutral	Unchanged	CS
32^*^	splicing (unassigned)	Bsol	Unknown	Unknown	CS
	p. Gly3191Arg/Gly3191	Bsol	Neutral to positive	Unchanged	CS
33	p. Thr4980Met/Thr4979	CTD	Unchanged	Hydrophilic to hydrophobic	CAC
	p. Gln4108His/Gln4109	EF1&2	Neutral to positive	Unchanged	CAC
	p. Pro4588Ser/Pro4587	pVSD	Unchanged	Hydrophobic to hydrophilic	CAC

*Pt, patient;*

* *, positive family history; CS, cytosolic shell; CAC, channel and activation core*.

**Figure 2:**
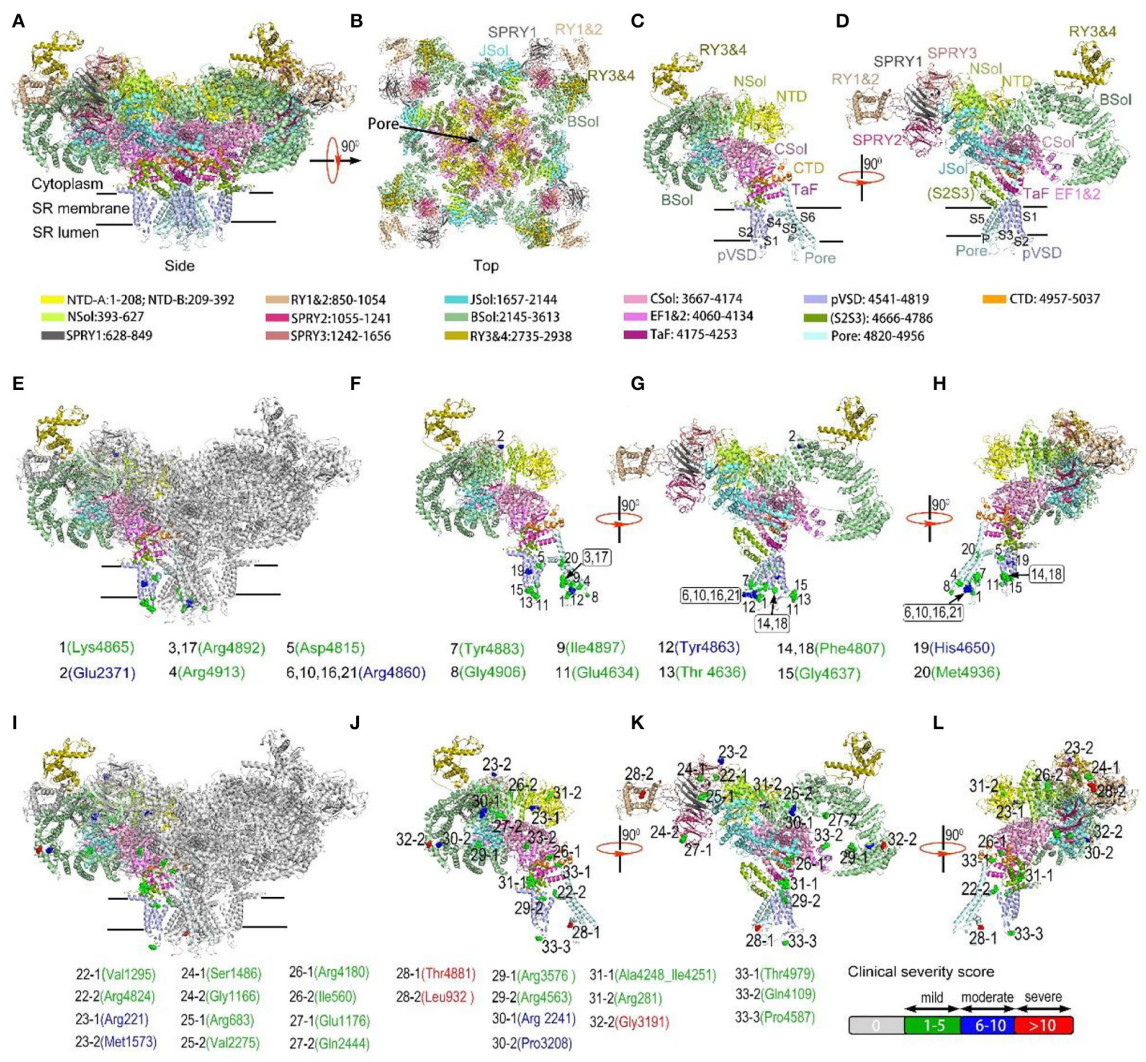
Variants mapping on the rabbit RyR1 monomers, presented with residues in rabbit matched to human variants. a-b, Structure of RyR1 tetramer from the membrane plane **(A)** and cytosolic plane **(B)**; **(C,D)** Structure of a single RyR1 monomer **(C)** and with 90-degree rotation **(D)**. Each domain was assigned a unique color. **(E)** Dominant RyR1 variants mapped on RyR1 tetramer with one monomer highlighted in color; **(F–H)** Dominant RyR1 variants on a single monomer **(F)**, with 90-degree **(G)** and 180-degree rotation **(H)**. **(I)** Recessive RyR1 variants mapped on RyR1 tetramer with one monomer highlighted in color; **(J–L)** Recessive RyR1 variants on a single monomer **(J)**, with 90-degree **(K)** and 180-degree rotation **(L)**. Each variant is represented by a colored sphere. Color coding for clinical severity was shown at the right bottom: mild 1-5 = green; moderate 6-10 = blue; severe >10= red. Arabic number before each residue designated the corresponding case number. Addictive number following case number “-1,” “-2” showing different recessive variants in the same case.

Affected domains of RyR1 were seen more clearly with variants mapping on the rabbit RyR1 monomers (Figure 2). Variants located in the transmembrane areas were mainly dominant, while most recessive variants were located in the top cytosolic area of RyR1. Dominant variants were predominantly clustered in the Pore areas, especially the S5-S6 loop and the P-helix, the key structural element of the central core (26). Out of the 45 variants, Arginine was the most frequently involved one (15/45, 7 dominant variants, eight recessive variants). Out of the 21 dominant variants, 11 had charge changes, four had hydrophilic property changes, one had both changes, and five variants had neither. Analysis of amino acid was not available in three recessive variations (two nonsense and one splice variation). Out of 22 recessive variants, 10 changed charges only, five changed hydrophilic property only, 7 neither. No recessive variant had both charge and the hydrophilic property changed.

**Figure 3:**
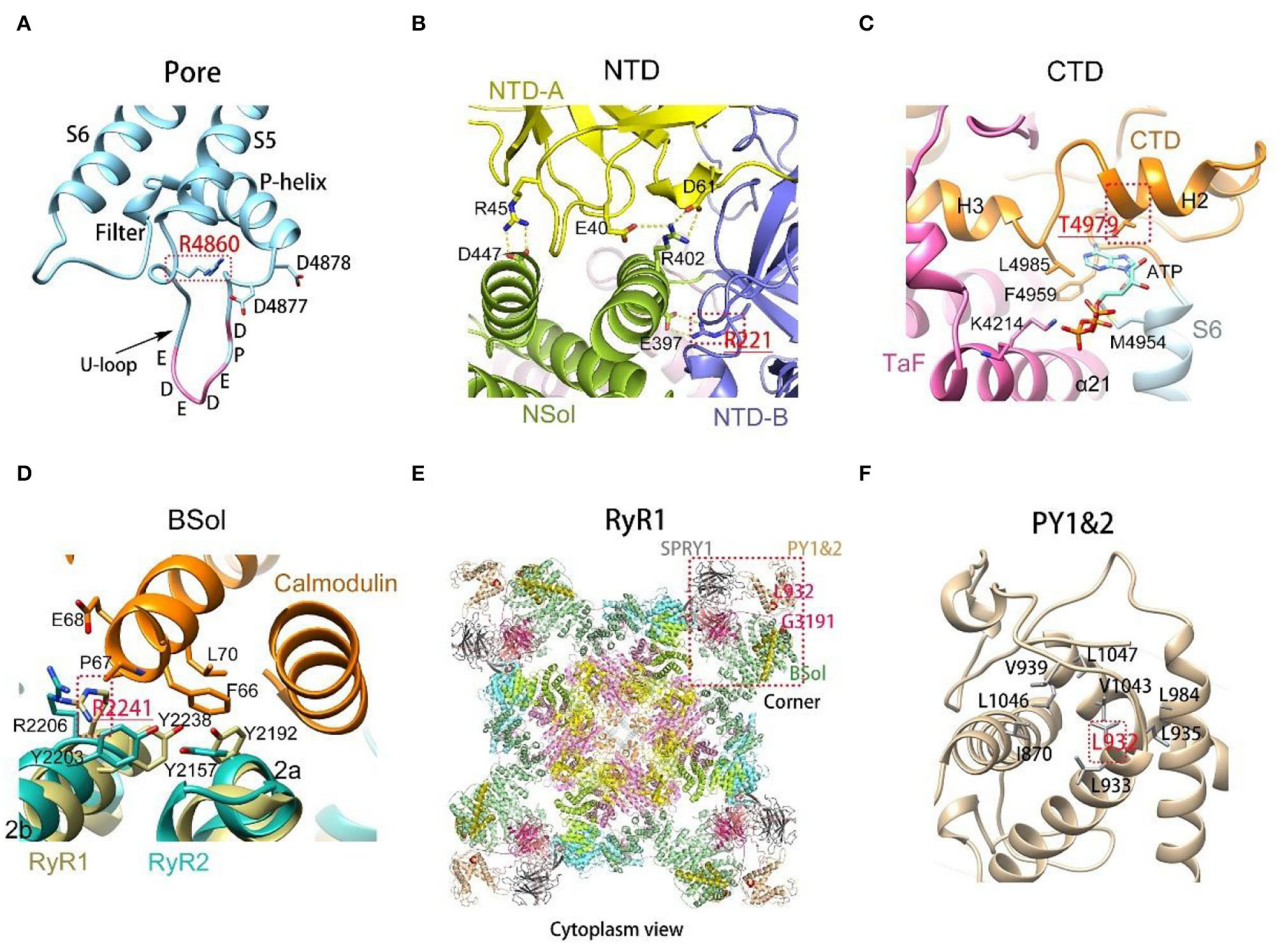
Three-dimensional structure of variants on rabbit RyR1 monomers, presented with residues in rabbit matched to human variants. **(A)** R4860 located on the U-motif of the S5-S6 loop, connecting with residues bring negative charges enriched in the U-motif; **(B)** view of the interactions among the three subunits of NTD. R221 on NTD-B connected with E397 on NSol; **(C)** T4979 located on the binding site of ATP, interfering with the combination between ATP and RyR1; **(D)** R2241 located on the binding site of calmodulin, interfering with the binding of Calmodulin to RyR1; **(E)** Gly3191 and Leu932 located on PY1&2 and the outermost part of Bsol, respectively, the presumed coupling sites among RyR1s; **(F)** the enlarged view of PY1&2, L931 located in the center of the domain, interacting with surrounding hydrophobic residues.

Detailed three-dimensional structure analysis of RyR1 variants was performed (Supplementary Table 5; Figure 3). The effect of variations was predicted based on the updated high-resolution structure of RyR1 (16). Most (15/21) dominant variants are located in areas with a relatively comprehensive understanding of the local 3D structures. The hydrogen and/or electrovalent bonds with adjacent residues were predicted to be broken in 10 patients, and the hydrophobic or hydrophilic interaction between residues was predicted to change in six patients, which would result in the instability of the local RyR1 structure. It is interesting to note that five out of seven dominant variants associated with moderate clinical severity were located on the S5-S6 loop, and 4 of them were located at the same residue Arg 4861 (Figure 3A). Compared to dominant variants, more recessive variants (8/25) are located in areas where high-resolution structure is unavailable now. Recessive variants may interfere with the function of RyR1 through different pathways: Variant p. Arg220Cys (Figure 3B) located on the NTD-B was predicted to interfere with the interaction between NTD-B and Nsol, which would result in the incomplete closure of the pore (27). Variant p. Thr4980Met (Figure 3C) located at the H2 helix of CTD, was predicted to interfere with the regulation of ATP on the function of RyR1 (28). Variant p. Arg 2241Ter (Figure 3D) located on the 2b helix of Bsol, was presumed to influence the binding of Calmodulin to RyR1 (29). It is interesting to note that the two deceased patients (Pt 28 and 32) both had one variant located in the area presumed to interfere with the coupling of RyR1. The 14ath helix of Bsol (the outermost of Bsol) where variant p. Gly3191Arg is located, and the PY1&2 helix where variant p. Leu931Pro is located (Figures 2k, 3E,F), were both regarded as the coupling sites of RyR1s according to the RyR two-dimensional (2D) pattern (30, 31).

## Discussion

Next generation sequencing has resulted in an explosion in the identification of rare novel variants of *RYR1*, considering the large size of the *RYR1* gene, and the frequency of *RYR1* variations in congenital myopathy. Cases with *RYR1* variations account for 33 to 59% of patients with congenital myopathies in large cohorts (2, 32). The clinical interpretation of these variants is becoming a great challenge, especially when multiple variants or *de novo* variants occur in patients with recessive inheritance mode. Although the ACMG guideline suggests evaluation of specific criteria (21), according to our experience, the assessment of the variant pathogenicity may be particularly challenging in the following clinical contexts:① A *de novo* variation was identified with the second variation in patients with negative family history. It is necessary to make sure that the two variants are trans or cis, which are not always available. Without functional study (which is not always available in clinical practice), it is difficult to confirm the inheritance mode and the pathogenicity of the variation. ②Three variations were identified in our Pt 33, one previously reported paternal likely pathogenic, two novel maternal variations with uncertain significance. Without functional study, it is unable to decide which maternal variant is the causing variation, which is why we put them both on the study.

The pathological heterogenicity of *RYR1*-related myopathy and its relationship with the location of *RYR1* variations in our patients were consistent with previous reports. Autosomal dominant *RYR1* variants for CCDs are enriched within the 3rd hotspot region (4), while recessive variations are dispersed along the whole gene, associated with varied forms of histopathological patterns including CNM, MmD, and fiber type disproportion (6–8, 33, 34), with core myopathy being the most frequent type (16). All dominant variations were missense, while recessive variations included missense and hypomorphic variations in our cohort. In a large cohort including 106 cases of recessive *RYR1-* related myopathies, nearly 50% of the cases had non-core myopathy, and hypomorphic variations were more frequently observed in non-central core myopathies (16). Patients with recessive *RYR1-* related non-core myopathies usually had a combination of a null variation and a missense variation (7, 8, 12, 35). Three of our patients with recessive truncate variations (2 nonsense, 1 splice variation) refused muscle biopsy. With only two non-core myopathies in our patients with recessive variations, it was difficult to analyze the relationship between variation type and pathology.

The relationship between clinical severity and *RYR1* genetic heterogenicity is complex. Clinical severity variance between patients with different inheritance mode was consistent with previous reports, that patients with recessive inheritance had a relatively more severe phenotype, with more severe bulbar and respiratory involvement in the neonatal period (3). It has been previously reported that hypomorphic variations were associated with more severe clinical manifestations (36). Patients with at least one hypomorphic allele had increased disease severity (16). Though no significant clinical severity variance was noted in our patients with two missense variations and with the combination of a missense and a hypomorphic variation. Considering no patient with two hypomorphic variations was noted, and the perinatal lethal phenotype of recessive RyR1 TM/Indel mouse model (37), we believe that the hypomorphic allele is related to more severe phenotypes.

Nearly all dominant variations (except one) were located in the Pore and pVSD domain, the key domains for the gating and activation of RyR1 (17). Dominant variants associated with moderately severe clinical manifestations clustered on the S5-S6 loop, around the P-helix. Comprising many residues with positive charges, the S5-S6 loop was regarded as an important binding site for junctional protein to RyR (38, 39). The P-helix contains many acidic residues which are expected to be anionic under physiological conditions (26). Arginine was the most frequently involved residue which brings a positive charge under physical conditions. Three-dimensional structure analysis of variants showed that hydrogen and/or electrovalent bond-breaking occur most frequently in dominant variants. Taking all those together, we presumed that changes in variant charges play a crucial role in the pathogenesis of dominant variants, which is consistent with previous research that abnormalities in ion conductance or ion selectivity play a crucial role in the pathogenesis of dominant *RYR1* variations (40).

Recessive variations affect more domains of RyR1 in our cohort. The most frequently involved domain was BSol, which is consistent with the previous report (20). Apart from that, SPRYs domains were also frequently involved in our cohort. The pathogenesis of recessive hypomorphic variations may due to the reduction of RyR1 protein. While the pathogenesis of recessive missense variations may vary with its locations on RyR1:① changes of intro-, intra-protomer interactions due to breaking of hydrogen and electrovalent bonds, ②interference between regulators and RyR1, just like variant p. Thr4980Met and p. Arg 2241Ter in our patients. RyR1 is regulated by many factors including Ca2+, Mg2+, ATP, calmodulin (CaM), protein kinases and phosphatases, and redox-active species (17). ③The possible co-effect of domains that contribute to the conformational changes of RyR1. The N-terminal of the bridging solenoid projecting toward the three SPRY domains has the calstabin-binding helix, which plays a critical role in stabilizing the Close state of RyR1, preventing pathologic intracellular calcium leak (26). ④Interference with the physical coupling between RyR1s. Intracellular Ca(2+) release was crucial for muscle contraction, which consists of brief Ca(2+) sparks. A single Ca (2+) spark formation needs 4 to 6 RyR1s activated synchronously (41, 42). Checkerboard arrays of RyR1 provide a structural basis for cooperative activation/deactivation of RyR1s through the process termed coupled gating (25). PY1&2 and the outermost part of Bsol were presumed to be the coupling sites (30, 31). It was interesting to note that variants p. Leu931Pro and p. Gly3191Arg in our study were presumed to take part in the coupling of RyR1, and patients with those variations had the most severe clinical manifestation (both died in early infancy). Our observation hints that the most harmful location of variants may be the sites involved in the coupling of RyR1, which is consistent with previous reports that the coupling of RyR1s plays a crucial role in the function of RyR1 (30, 41).

One limitation of the present study is that no functional study of variants was performed. The interpretation of the possible structural defects of the molecule is solely predicted based on current knowledge about the structure and the function of RyR1. Though transgenic mouse model of *RYR1*- related myopathy has been successfully constructed for more than 10 years (43). The structure of RyR1 is usually analyzed with purified protein obtained from rabbits (25). Structural or functional analysis of RyR1 variants in animal models will be more helpful to understand the defects caused by gene variations.

## Conclusion

The present study expanded our understanding of *RYR1*-related myopathy, with corroboration of the phenotype, genotype, and protein structure of *RYR1*. Clinical diversity of *RYR1*-related myopathies with different inheritance mode was observed. Missense was the most common variation type regardless of inheritance mode. Arginine was the most frequently involved residue. Variant mapping showed that dominant variants mainly clustered in channel Pore and Pseudo-voltage-sensor domain, while Bsol and SPRYs were frequently involved in key domains in recessive variations. Detailed analysis of the high-resolution three-dimensional structure of variants implied that dominant variants affect the conduction of the pore mainly by breaking the hydrogen or electrovalent bonds between residues, while recessive variants may impact the function of RyR1 through different pathways. Variants interfering with RyR1 coupling may cause the most severe clinical manifestation.

## Data Availability Statement

The datasets presented in this study can be found in online repositories. The names of the repository/repositories and accession number(s) can be found in the article/Supplementary Materials.

## Ethics Statement

The studies involving human participants were reviewed and approved by the Ethics Committee of Peking University First Hospital (Beijing, China, approved number 2018-265). Written informed consent to participate in this study was provided by the participants' legal guardian/next of kin.

## Author Contributions

XC and RW: had full access to all of the data, take responsibility for the accuracy of the data analysis, and drafted and revised the paper. CW, JL, LQ, and HY: acquisition, analysis, and interpretation of clinical and pathologic data. YM, ZW, and HX: analysis of genetic data. RW: analysis of protein structure. ZW and HX: provided comments and revised the paper. All authors contributed to the article and approved the submitted version.

## Conflict of Interest

The authors declare that the research was conducted in the absence of any commercial or financial relationships that could be construed as a potential conflict of interest.

## Publisher's Note

All claims expressed in this article are solely those of the authors and do not necessarily represent those of their affiliated organizations, or those of the publisher, the editors and the reviewers. Any product that may be evaluated in this article, or claim that may be made by its manufacturer, is not guaranteed or endorsed by the publisher.
