# Supplementary material for: Correlation of Phenotype–Genotype and Protein Structure in RYR1-Related Myopathy
Source: Front Neurol. 2022 May 26;13:870285. doi: 10.3389/fneur.2022.870285 (PMC9178086; doi:10.3389/fneur.2022.870285)
Supplement: Supplementary file 1 [file Data_Sheet_1.PDF]

Table S1 Disease severity rating scale

Item	Severity score				
	0	1	2	3	4
Onset age	≥1 year	< 1 year	/	/	
Perinatal asphyxia	No	Yes	/	/	/
Feeding difficulty	No	Yes, no need of tube feeding	Tube feeding	/	/
Respiratory insufficiency	No	Yes, without ventilator support	Ventilator support	/	/
Congenital hip dysplasia	No	Yes	/	/	/
Motor development	Normal	Attain independent walking before 2 years old	Attain independent walking at 2≤3 years old	Attain independent walking at 3≤4 years old	Fail to walk alone at >4 years old
Infancy survival	Yes	/	/	/	Death
