# Supplementary material for: Correlation of Phenotype–Genotype and Protein Structure in RYR1-Related Myopathy
Source: Front Neurol. 2022 May 26;13:870285. doi: 10.3389/fneur.2022.870285 (PMC9178086; doi:10.3389/fneur.2022.870285)
Supplement: Supplementary file 2 [file Data_Sheet_2.PDF]

Table S2 Gene panel for hereditary muscle disease (the 169 genes)

ABHD5	ACADVL	ACTA1	ACVR1	AGK	AGL	AGRN	ALG13
ALG14	ALG2	ANO5	ATP2A1	ATP5A1	B3GALNT2	B3GNT1	BAG3
BIN1	CABC1	CACNA1A	CACNA1S	CAPN3	CAV3	CCDC78	CFL2
CHAT	CHKB	CHRNA1	CHRNB1	CHRND	CHRNE	CHRNG	CHST14
CLCN1	CNBP	SLC28A2	CNTN1	COL12A1	COL6A1	COL6A2	COL6A3
COLQ	CPT2	CRYAB	DAG1	DARS	DES	DMD	DMPK
DNAJB6	DNM2	DOK7	DOLK	DPAGT1	DPM1	DPM2	DPM3
DUX4	DYSF	ECEL1	EMD	ENO3	ETFA	ETFB	ETFDH
FHL1	FKBP14	FKRP	FKTN	FLNC	GAA	GBE1	GFPT1
GMPPB	GNE	GTDC2	GYG1	GYS1	HADHB	HSPG2	ISCU
ISPD	ITGA7	ITGA9	KBTBD13	KCNA1	KCNE3	KLHL40	KLHL41
KLHL9	LAMA2	LAMB2	LAMP2	LARGE	LDB3	LDHA	LMNA
LPIN1	LRP4	MAMLD1	MATR3	MEGF10	MSTN	MTM1	MTMR14
MUSK	MYBPC1	MYBPC3	MYF6	MYH14	MYH2	MYH7	MYOT
NDUFB3	NEB	PABPN1	PFKM	PGAM2	PGK1	PGM1	PHKA1
PIEZO2	PLEC	PLOD1	PLOD2	PLOD3	PNPLA2	POLG2	POMGNT1
POMK	POMT1	POMT2	PRKAG2	PTPLA	PTRF	PUS1	PYGM
RAPSN	RBCK1	RYR1	SCN4A	SEPN1	SGCA	SGCB	SGCD
SGCE	SGCG	SGK196	SIL1	SLC16A2	SLC22A5	SLC25A20	SMCHD1
SYNE1	SYNE2	TCAP	TIA1	TK2	TMEM43	TMEM5	TNNT1
TNPO3	TOR1AIP1	TPM2	TPM3	TRAPPC11	TRIM32	TTN	VCP
YARS2							
