# Supplementary material for: Correlation of Phenotype–Genotype and Protein Structure in RYR1-Related Myopathy
Source: Front Neurol. 2022 May 26;13:870285. doi: 10.3389/fneur.2022.870285 (PMC9178086; doi:10.3389/fneur.2022.870285)
Supplement: Supplementary file 3 [file Data_Sheet_3.PDF]

Table S3 Clinical feature of patients with *RYR1*-related myopathy

Pt/ Sex	Onset age (m)	Reduced fetal movement	Asphyxia at birth	Feeding difficulty (infancy)	Ventilator support (infancy)	Congenital hip dysplasia	Independent walking (m)	Age at last visit (y)	Motor ability at last visit	Muscular complication	Clinical severity score
1/F	24	+	-	-	-	-	48	20	Wheelchair	+	3
2/F	Birth	+	-	+	-	-	Never	4.5	Stand with support	+	6
3/F*	12	-	-	-	-	-	20	24	Ambulant	-	2
4/M*	Birth	-	-	-	-	+	Never	9	Wheelchair	+	5
5/M	Birth	+	-	-	-	+	19	4	Ambulant	-	3
6/F	Birth	/	-	+	-	+	Never	3.5	Stand with support	+	6
7/M	6	/	-	-	-	-	48	19	Ambulant	-	4
8/M*	12	-	-	-	-	-	16	8	Ambulant,	-	2
9/F	Birth	-	-	-	-	+	36	4	Ambulant	-	4
10/M	Birth	+	-	-	-	+	Never	2.3	Stand with support	+	5
11/M*	12	-	-	-	-	-	14	42	Ambulant	-	2
12/M*	Birth	+		+	-	+	Never	1.5	Stand with support	+	6
13/F*	18	-	-	-	-	-	24	34	Ambulant	+	1
14/M	6	+	-	+	-	-	30	4	Ambulant	-	4
15/F	24	+	-	-	-	-	12	27	Ambulant	-	1
16/F	Birth	+	-	+	-	+	Never	2	Stand with support	-	5
17/F*	Birth	+	+	-	-	+	Never	1.5	Sit	-	3
18/M*	Birth	+	-	-	-	+	Never	0.1	/	-	3

Pt/ Sex	Onset age (m)	Reduced fetal movement	Asphyxia at birth	Feeding difficulty (infancy)	Ventilator support (infancy)	Congenital hip dysplasia	Independent walking (m)	Age at last visit (y)	Motor ability at last visit	Muscular complication	Clinical severity score
19/F	Birth	+	+	+	+	+	Never	2	Sit	+	9
20/F	18	/	-	-	-	+	14	25	Ambulant	-	2
21/F	6	+	-	-	-	+	Never	6.1	Stand with support	+	6
22/M	12	-	-	-	-	-	14	16	Ambulant	-	2
23/M*	Birth	-	-	+	+	-	24	5	Ambulant	+	6
24/M	Birth	+	-	+	-	-	12	17	Ambulant	+	3
25/M	12	-	-	-	-	-	12	18	Ambulant	-	2
26/F	6	-	-	-	-	+	24	21	Ambulant	-	3
27/M	Birth	-	-	-	-	-	13	16	Ambulant	-	2
28/F	Birth	+	+	+	+	+	Never	0.6	Death	-	11
29/F	12	-	-	-	-	-	18	9	Ambulant	+	2
30/M	Birth	-	-	+	+	-	Never	1	Head control	-	6
31/M	12	-	-	-	-	+	32	5	Ambulant	-	4
32/M*	Birth	/	+	+	+	-	Never	0.1	Death	/	10
33/M	4	-	-	-	-	-	16	7	Ambulant	-	2

Note: /, no data; +, present; –, absent; *, positive family history; M, male; F, female; y, year; m, month;
