# Supplementary material for: Correlation of Phenotype–Genotype and Protein Structure in RYR1-Related Myopathy
Source: Front Neurol. 2022 May 26;13:870285. doi: 10.3389/fneur.2022.870285 (PMC9178086; doi:10.3389/fneur.2022.870285)
Supplement: Supplementary file 4 [file Data_Sheet_4.PDF]

Table S4 Data of novel *RYR1* variations (submitted to ClinVar)

Pt	Inheritance	Variation	Protein	Pathogenicity	Frequency in gnomAD (exome_ALL)	Frequency in gnomAD (exome_EAS)	ClinVar Allele ID	Accession number
7	AD	c.14650T>C	p.Tyr4884His	LP	-	-	1341368	VCV001341368.1
11*	AD	c.13904A>G	p.Glu4635Gly	LP	-	-	590441	VCV000590441.2
12*	AD	c.14591A>C	p.Tyr4864Ser,	LP	-	-	1341372	VCV001341372.1
20	AD	c.14811C>G	p.Ile4937Met	LP	-	-	1341373	VCV001341373.1
28	AR	c.2792 T>C	p. Leu931Pro	LP	-	-	1341374	VCV001341374.1
29	AR	c.10729C>G,	p.Gln3577Gly	LP	-	-	1341375	VCV001341375.1
		c.13691G>A	p.Arg4564Gln	LP	0.00003187	0	218466	VCV000218466.6
30	AR	c.9623C>T	p.Pro3208Leu	LP	0.00003185	0	586415	VCV000586415.5
31		c.12739_12750del	p.Ala4247_Ile4250del	Uncertain	-	-	1341371	VCV001341371.1
		c.839G>A	p.Arg280Gln	LP	-	-	861815	VCV000861815.3
32*	AR	c.7614+1G>A	Splicing	Pathogenic	-	-	1341369	VCV001341369.1
		c.9571G>A	p.Gly3191Arg	LP	-	-	436627	VCV000436627.6
33	AR	c.12324G>C	p.Gln4108His	Uncertain	0.0001	0.0026	1011341	VCV001011341.2
		c.13762C>T	p.Pro4588Ser	Uncertain	-	-	1341370	VCV001341370.1

Note: AD, autosomal dominant; AR, autosomal recessive; LP, likely pathogenic; exome_ALL, exome examination data in people of all ethnic group,: exome_EAS, exome examination data in east Asian people; -, not included.
