# Supplementary material for: Correlation of Phenotype–Genotype and Protein Structure in RYR1-Related Myopathy
Source: Front Neurol. 2022 May 26;13:870285. doi: 10.3389/fneur.2022.870285 (PMC9178086; doi:10.3389/fneur.2022.870285)
Supplement: Supplementary file 5 [file Data_Sheet_5.PDF]

Table S5 Three-dimensional structure analysis of RyR1 variants (based on the updated structure of RyR1 in rabbit(1))

Pt	Protein human/rabbit	Spatial structure of wild amino acid	Possible effect of variant	Clinical severity
1	p. Lys4866Gln /Lys4865	Located on the U-motif of the S5-S6 loop, connecting with adjacent Asp4873 and Glu4867 by electrostatic interaction	Electrostatic interaction predicted to be replaced by hydrogen bond, impacting the combination between RyR1 and junctional protein (2, 3)	Mild
2	p. Glu2371Lys /Glu2371	Located between the 5a helix and 5a` helix of Bsol, adjacent to NTD-A. High-resolution structure of the site is unavailable so far.	Predicted to interfere with the connection between Bsol and NTD-A	Moderate
3 *	p. Arg4893Gln /Arg4892	Located on P-helix, connecting with Tyr4888 on the P-helix by the hydrogen bond	Predicted to impact the stability of P-helix.	Mild
4 *	P. Arg4914Thr/Arg4913	Located at the N terminal of S6, connecting with Asp4917 on S6 by the electrovalent bond	Electrovalent bond with Asp4917 predicted to be replaced by the hydrogen bond, impacting the stability of the N terminal of S6.	Mild
5	p. Asp4816Gly/Asp4815	Located at the C terminal of S4, in the “pocket” of pVSD, connecting with Arg4563 (on S1) in the “pocket” by the electrovalent bond. It is the binding site of small molecules such as diamide	Predicted to impact the stability of the “pocket” of pVSD.	Mild
6	p. Arg4861His /Arg4860	Located on the U-motif of the S5-S6 loop, connecting with Asp4877 on the other side of the U-motif by the electrovalent bond	Electrovalent bond predicted to be broken, impacting the stability of the U-motif, and the combination between RyR1 and junctional protein (2, 3)	Moderate
7	p. Tyr4884His/Tyr4883	Located on P-helix, connecting with Tyr4849、Val4848, Ala4845 on S5 by hydrophobic interaction	Hydrophobic interactions between residues predicted to disappear, impacting the stability of P-helix	Mild
8 *	p. Gly4907Ser/Gly4906	Located on the loop between S6 and the	The variant predicted to build connection	Mild

		selective filter	with Glu4910 on the loop by the hydrogen bond, changing the structure of the selective filter	
9	p. Ile4898Thr/Ile4897	Located on the selective filter, connecting with Val4889, Phe4885 on the P-helix, and Phe4916 、 Phe4920 on S6 by hydrophobic interaction	Hydrophobic interactions between the residues predicted to disappear, impacting on the stability of the selective filter	Mild
10	p. Arg4861Cys/Arg4860	Located on the U-motif of the S5-S6 loop, connecting with Asp4877 on the other side of the U-motif by the electrovalent bond	Electrovalent bond predicted to be broken, impacting the stability of the U-motif, and the combination between RyR1 and junctional protein (2, 3)	Moderate
11*	p. Glu4635Gly/Glu4634	Located on the S1-S2 loop of pVSD, high-resolution structure of the site is unavailable so far.	Presumed to impact the combination between RyR1 and junctional protein (,2,3)	Mild
12*	p. Tyr4864Ser/Tyr4863	Located on the U-motif of the S5-S6 loop, connecting with Phe4885 on P-helix, and Ile4897, Ile4901 on the selective filter by hydrophobic interaction; connecting with His4886 on P-helix, Arg4913 on S6 with the hydrogen bond	Hydrophobic interaction between residues predicted to disappear, hydrogen binding predicted to be weakened, impacting the stability of the U-motif, and the combination between RyR1 and junctional protein (2,3)	Moderate
13*	p. Thr4637Ala/Thr4636	Located on the S1-S2 loop of pVSD, high-resolution structure of the site is unavailable so far	Presumed to impact the combination between RyR1 and junctional protein (2,3)	Mild
14	p. Phe4808Asn/Phe4807	Located on the N terminal of S4, adjacent to the S3-S4 loop, connecting with Leu4577 on S1， Tyr4580, Tyr4629 on the S1-S2 loop by hydrophobic interaction	Hydrophobic interaction among residues predicted to disappear, impacting the combination between RyR1 and junctional protein (2,3)	Mild
15	p. Gly4638Asp/Gly4637	Located on the S1-S2 loop of pVSD,	Presumed to impact the combination	Mild

		high-resolution structure of the site is unavailable so far	between RyR1 and junctional protein (2,3)	
16	p. Arg4861His/Arg4860	Located on the U-motif of the S5-S6 loop, connecting with Asp4877 on the other side of the U-motif by the electrovalent bond	Electrovalent bond predicted to be broken, impacting the stability of the U-motif, and the combination between RyR1 and junctional protein (2,3)	Moderate
17 *	p. Arg4893Gln/Arg4892	Located on P-helix, connecting with Tyr4888 on P-helix by the hydrogen bond	Predicted to impact the stability of P-helix.	Mild
18*	p. Phe4808delinsAsn/ Phe4807	Located on the N terminal of S4, adjacent to the S3-S4 loop, connecting with Leu4577 on S1, Tyr4580, Tyr4629 on the S1-S2 loop by hydrophobic interaction	Hydrophobic interaction among residues predicted to disappear, impacting the combination between RyR1 and junctional protein (2,3)	Mild
19	p. His4651Pro/His4650	Located on the middle transmembrane part of S2, the center of pVSD, connecting with Ser4799 on S3, His4812 on S4 by hydrophilic interaction	Hydrophilic interaction among residues predicted to disappear, interfering with the conformational change of the pVSD	Moderate
20	p. Ile4937Met/Ile4936	Located at the middle part of S6, close to the gate (Ile4937)	Presumed to interfere with the conformational change of the gate	Mild
21	p. Arg4861Cys/Arg4860	Located on the U-motif of the S5-S6 loop, connecting with Asp4877 on the other side of the U-motif by the electrovalent bond	Electrovalent bond predicted to be broken, impacting the stability of the U-motif, and the its combination between RyR1 and junctional protein (2,3)	Moderate
22	p. Val1294Phe/Val1295	Located on the top outside loop of SPRY3, high-resolution structure of the site is unavailable by now	Predicted to interfere with the interaction between RyR1 and DHPR(4)	Mild
	p. Arg4825Cys/Arg4824	Located at the S4-5 linker helix, adjacent to S4, the side chain of Arg reaching to the pocket of pVSD,	Predicted to interfere with the interaction between pVSD and the S4-5 linker	

23*	p. Arg220Cys/Arg221	Located on the N-TDB，connecting with Glu397 on Nsol by the electrovalent bond	Electrovalent bond predicted to be broken, interfering with the interaction between N-TDB and Nsol	Moderate
	p. Met1572Thr/Met1573	Located on the top of SPRY3, high-resolution structure of the site is unavailable by now	Hydrophobic Met predicted to be replaced by hydrophilic Thr, interfering with the interaction between RyR1 and DHPR(4)	
24	p. Ser1485Asn/Ser1486	Located on the top outside loop of SPRY3, high-resolution structure of the site is unavailable by now	Predicted to interfere with the interaction between RyR1 and DHPR(4)	Mild
	p. Gly1165Asp/Gly1166	Located on SPRY2, adjacent to the 17b helix of Bsol	Predicted to build connection with Asn3428 on 17b helix by the hydrogen bond, interfering with the interaction between Bsol and SPRY2	
25	p. Arg682Gly/Arg683	Located on SPRY1, connecting with Asp 709 in the center of SPRY1 by electrostatic interaction, adjacent to the binding site of FKBP12 (Phe674)	Electrostatic interaction predicted to be broken, impacting the stability of SPRY1, interfering with the binding of KKBP12 to RyR1 (5)	Mild
	p. Val2275Cys/Ser2275	Located on the 3b helix of Bsol, connecting with Leu2236 on 2b helix by hydrophobic interaction, where is the binding site of Calmodulin	Hydrophobic interaction with Leu2236 predicted to disappear, impacting the stability of 2b helix, interfering with the binding of Calmodulin to RyR1	
26	p. Arg4179His/Arg4180	Located at the β-sheep structure of TaF, connecting with Glu4981 on H2 helix of CTD by electrostatic interaction.	Electrostatic interaction predicted to be broken, interfering with the interactions between TaF and CTD	Mild
	p. Ile559AsnfsTer11/Ile560	Located on the 8 helix of Nsol, connecting with Tyr523 on the 5 helix, Val547 on the 7 helix by hydrophobic interaction	Hydrophobic interaction predicted to disappear, impacting the stability of Nsol	

27	p. Glu1175Lys/Glu1176	Located on SPRY2, adjacent to the 17b and the 20 helix of Bsol, high-resolution structure of the site is unavailable by now	Charge changes with variant presumed to interfere with the interaction between SPRY2 and Bsol	Mild
	p. Gln2444Ter/Gln2444	Located on the loop of 5b-6a helix of Bsol, high-resolution structure of the site is unavailable by now	The synthetic stop of amino acid presumed to impact the stability of Bsol	
28	p. Thr4882Met/Thr4881	Located at P-helix, connecting with Asp4878, Asp4877 on the U-motif of the S5-S6 loop by the hydrogen bond	Hydrogen bond predicted to be broken, impacting the stability of the U-motif, and the combination between RyR1 and junctional protein (2, 3)	Severe
	p. Leu931Pro/Leu932	Located at PY1&2 helix, connecting with adjacent Ile870, Leu933, Val939, Leu984, Val1043, Leu1046, Leu1047 by hydrophobic interaction	Predicted to interfere with the coupling among RyR1s (PY1&2 was supposed to be the coupling site between RyRs) (5,6)	
29	p. Arg3576Gly/Arg3576	Located on the C terminal of the 24 helix of Bsol, high-resolution structure of the site is unavailable by now	The interaction with other residues presumed to be broken, impacting the stability of local structure	Mild
	p. Arg4563Gln/Arg4563	Located at the cytosolic side of S1 helix, in the pocket of pVSD, connecting with Tyr4791 on S3 by the hydrogen bond	Hydrogen bond predicted to minimized, impacting the stability of the pocket structure of pVSD	
30	p. Arg2241Ter/Arg2241	Located on the 2b helix of Bsol, adjacent to Jsol.	Presumed to be the binding site of Calmodulin. The synthetic stop of amino acid predicted to impact the binding of Calmodulin to RyR1 (6)	Moderate
	p. Pro3208Leu/Pro3208	Located on the 14b helix of Bsol, connecting with Leu3194 and Leu3197 on 14a helix by hydrophobic interaction	Hydrophobic interactions predicted to disappear, indirectly impacting the stability of the 14a helix of Bsol and the	

			coupling between RyR1s (6,7)。	
31	p. Ala4247_Ile4250del/Ala4248_Ile4251	Located at the α22 helix of the C terminal of TaF, Ile4247(α22)connecting with Lue4668（on S2`）, Leu4664（on S2）by hydrophobic interaction, Ile4251 connecting with Leu4664（on S2） by hydrophobic interaction	Hydrophobic interactions predicted to disappear, interfering with the interactions between TaF and S2S3	Mild
	p. Arg280Gln/Arg281	Located at NTB-B, adjacent to the interface between NTD-B and NTD-A,	Change of charges predicted to interfere with the interaction between NTD-B and NTD-A	
32*	Splicing (unassigned)	Located on Bsol, with no residue can be assigned	Unable to predict	Severe
	p. Gly3191Arg/Gly3191	Located on the 14a helix of Bsol, the outermost part of Bsol. high-resolution structure of the site is unavailable by now	Presumed to directly interfere with the coupling between RyR1s(7, 8)	
33	p. Thr4980Met/Thr4979	Located at H2 helix of CTD, taking part in the binding of ATP through hydrophilic action	Hydrophilic action predicted to disappear, interfering with the binding of ATP to RyR1 (9)	Mild
	p. Gln4108His/Gln4109	Located at E2 helix of EF1&2, connecting with Gln4094 on F1 helix by the hydrogen bond	Hydrogen bond predicted to minimize, impacting the stability of EF1&2	
	p. Pro4588Ser/Pro4587	Located on the S1-S2 loop of pVSD, high-resolution structure of the site is unavailable by now	Hydrophobic action predicted to disappear, impacting the stability of the S1-S2 loop, and the combination between RyR1 and junctional protein (2,3)	

Note: *, positive family history; Mild, clinical severity score 0-5; Moderate, clinical severity score 6-10; Severe, clinical severity score >10

Reference:

1. Meissner G. The structural basis of ryanodine receptor ion channel function. *J Gen Physiol.* (2017) 149:1065-1089. doi: 10.1085/jgp.201711878
2. Beard NA, Wei L, Dulhunty AF. Ca(2+) signaling in striated muscle: the elusive roles of triadin, junctin, and calsequestrin. *Eur Biophys J.* (2009) 39:27-36. doi: 10.1007/s00249-009-0449-6
3. Zhang L, Kelley J, Schmeisser G, Kobayashi YM, Jones LR. Complex formation between junctin, triadin, calsequestrin, and the ryanodine receptor. Proteins of the cardiac junctional sarcoplasmic reticulum membrane. *J Biol Chem.* (1997) 272:23389-23397. doi: 10.1074/jbc.272.37.23389
4. Bai XC, Yan Z, Wu J, Li Z, Yan N. The Central domain of RyR1 is the transducer for long-range allosteric gating of channel opening. *Cell Res.* (2016) 26:995-1006. doi: 10.1038/cr.2016.89
5. Zalk R, Clarke OB, des Georges A, Grassucci RA, Reiken S, Mancia F *et al.* Structure of a mammalian ryanodine receptor. *Nature.* (2015) 517:44-49. doi: 10.1038/nature13950
6. Gong D, Chi X, Wei J, Zhou G, Huang G, Zhang L *et al.* Modulation of cardiac ryanodine receptor 2 by calmodulin. *Nature.* (2019) 572:347-351. doi: 10.1038/s41586-019-1377-y
7. Yin CC, Blayney LM, Lai FA. Physical coupling between ryanodine receptor-calcium release channels. *J Mol Biol.* (2005) 349:538-546. doi: 10.1016/j.jmb.2005.04.002
8. Yin CC, Han H, Wei R, Lai FA. Two-dimensional crystallization of the ryanodine receptor Ca2+ release channel on lipid membranes. *J Struct Biol.* (2005) 149:219-224. doi: 10.1016/j.jsb.2004.10.008
9. des Georges A, Clarke OB, Zalk R, Yuan Q, Condon KJ, Grassucci RA *et al.* Structural Basis for Gating and Activation of RyR1. *Cell.* (2016) 167:145-157.e117. doi: 10.1016/j.cell.2016.08.075
